# The Food Bill at St. George's

**Published:** 1916-04-08

**Authors:** 


### HOSPITAL EXPENDITURE IN WAR TIME.

### The Food Bill at St. George's.

The annual reports of the large voluntary hospitals in London will not be ready for publication for some considerable time, and consequently the precise effect of the war on hospital expenditure will not be common knowledge for at least a month or six weeks from now. It can be said positively, however, that the accounts will show a large increase on the expenditure side, for no efforts to economise can counteract the effect of the rising cost of foodstuffs and of drugs and medical and surgical appliances which has been steadily going on since the outbreak of the war. Through the courtesy of the authorities at St. George's Hospital the writer has been furnished with some details of the accounts of that institution for 1915, and if it can be assumed—as it probably can—that the experiences at other large establishments have been much the same as at St. George's, it will not be necessary to await the publication of the annual reports to obtain a fair idea of the extra cost that was entailed by a year of war-time prices.

### £4,000 Increase in Total Expenditure.

The average annual expenditure at St. George's Hospital in recent years has been about £40,000. In 1914 the average was exceeded by £1,576, and to a certain extent this increase was due to the higher cost of commodities, for 1914 included five war months in which prices had already begun to advance rapidly. The accounts for 1915—a full twelve months of war—show that the total amount expended was approximately £4,000 in excess of 1914, and the extent to which this excess was due to the rise in the cost of foodstuffs was precisely £2,524 18s. 4d. When the increase in the domestic accounts (coal, etc.) is added to this total it will be found that practically the whole of the excess of total expenditure is accounted for. The expenses of the surgery and dispensary have not increased during the year, chiefly owing to the policy of making large purchases in bulk at the beginning of the year.

The particulars furnished by the authorities enable us to publish the following interesting table, which shows clearly which items in the food bill are most responsible for the increase :

| | 1914. | £ s. d. | 1915. | £ s. d. |
|---|---|---|---|---|
| 1. Meat ... ... | | 2,698 19 8 | | 3,423 8 4 |
| 2. Fish ... | £639 5 4 | | £673 16 3 | |
| Poultry | 355 8 7 | | 564 19 8 | |
| | | 994 13 11 | | 1,238 15 11 |
| 3. Butter, cheese, etc. | | 1,396 14 6 | | 1,720 17 4 |
| 4. Eggs ... ... ... | | 532 15 7 | | 515 4 6 |
| 5. Milk ... ... ... | | 1,692 1 4 | | 1,762 15 0 |
| 6. Bread, flour, etc. ... | | 700 11 11 | | 1,055 6 3 |
| 7. Grocery ... ... | | 1,076 9 7 | | 1,712 14 6 |
| 8. Vegetables and fruit | | 438 0 1 | | 481 11 1 |
| 9. Malt liquors ... | | 142 14 1 | | 240 12 2 |
| 10. Aërated waters and ice ... ... ... | | 150 11 2 | | 197 4 3 |
| | | £9,823 11 10 | | £12,348 9 4 |

From a simple calculation it is seen that while the butcher's bill shows the largest gross increase there is a much bigger percentage increase in some of the other items. Thus in the grocery account, out of an expenditure of £1,712 14s. 6d. the sum of £636 4s. 11d. is due to additional charges. Even in the item "Butter, cheese, etc.," there is an increase of £324 3s. 10d., notwithstanding that, at any rate during part of the year, margarine was substituted for butter, although butter has always been supplied to the military patients. The only item showing a decrease is eggs. At first sight this may seem curious, when it is remembered that throughout the whole of 1915 almost famine prices had to be paid for eggs. The decrease does not mean that fewer eggs were supplied to the patients; on the contrary, a greater number of eggs was consumed, but a large proportion was supplied by generous donors. Indeed, the contributions of consumable commodities have not been restricted to eggs, but consignments of all sorts of foodstuffs—generally those which are classified as luxuries, were presented to the hospital in the course of the year. Had it not been for these donations the increase in the food bill would have been materially greater, and this uncertain factor would render an exact calculation of the effect of the war on hospital expenditure very difficult. Again, it would be incorrect to assume that the total increase as shown above is wholly due to advanced prices, for the admission of military patients into the wards last year would clearly necessitate the purchase of a larger volume of commodities. But taking all factors into the calculation it may be stated that the extent to which the increase in the food bill at St. George's Hospital last year was due to the rise in the cost of foods was between 20 and 25 per cent. This figure would agree approximately with the percentage increase that has taken place since the beginning of last year in the prices of foods in large towns as shown by the Board of Trade figures. The percentage increase on normal pre-war prices as compared with those now ruling works out at 49 per cent., but a calculation based on a comparison between prices at the end of 1914 and the end of 1915 shows that during the twelve months the increase was about 25 per cent.

### Treatment of Wounded Soldiers.

Like every other voluntary hospital in the country, St. George's has felt the effect of the war in other ways than in the increased prices of provisions. On the outbreak of hostilities the authorities allotted a hundred beds for the accommodation of naval and military cases, and although at the present time only forty-six of these beds are actually in occupation, the arrangements are such that the full hundred are available whenever required. It is a curious fact, but a source of gratification, to find that, although the total accommodation has only been increased by eighteen beds, the waiting list for civil patients is much lighter than at a normal time. This is due to a variety of influences arising directly and indirectly out of the war, and to one or other of these same causes is due the fact that the proportion of female to male civilian patients under treatment in the hospital has so changed that two male wards of twenty-five beds have been converted into female wards. There has also been a falling-off in the number of out-patients, especially among males, and while there have been fewer drunken cases the casualty department has been much busier at night in consequence of accidents due to the darkness of the busy thoroughfares in the neighbourhood of the hospital.

Like other of the large London institutions, St. George's has provided special facilities for the treatment of would-be recruits who are unfit for service because of some defective condition that is amenable to treatment. Thus many cases of hernia and varicocele have been treated, and in this way the hospital has augmented His Majesty's Forces by making men fit for the Army who before treatment had been rejected. Then another of the many ways in which the hospital has contributed to the common cause is by the inoculation against typhoid and paratyphoid of British and French Red Cross workers, of whom 263, chiefly motor drivers, have been inoculated with triple vaccine.

Schemes in Abeyance.

Such schemes as the authorities had in contemplation for the development of various departments have for the most part been abandoned for the time being. Improvements, however, have been effected in the $x$-ray department, while great alterations have been made in the system under which massage was practised. Formerly massage was done in the out-patients' theatre, a most inconvenient arrangement owing to cramped space, and a much-needed alteration has been made by converting two rooms in the basement which were formerly used by the steward into massage-rooms, while a small building has been erected in the yard for the use of the steward. It has, of course, been impossible to proceed with the plans for the new out-patients' casualty departments and the new nurses' home, although it is hoped to proceed with this scheme as soon as the times are favourable.

